# Prevention of Nausea and Vomiting in Patients Undergoing Oral Anticancer Therapies for Solid Tumors

**DOI:** 10.1155/2015/309601

**Published:** 2015-09-03

**Authors:** Ana Lúcia Costa, Catarina Abreu, Teresa Raquel Pacheco, Daniela Macedo, Ana Rita Sousa, Catarina Pulido, António Quintela, Luís Costa

**Affiliations:** ^1^Oncology Division, Hospital de Santa Maria, Centro Hospitalar de Lisboa Norte, EPE, Avenida Professor Egas Moniz, 1649-035 Lisbon, Portugal; ^2^Instituto de Medicina Molecular, Faculdade de Medicina da Universidade de Lisboa, Avenida Professor Egas Moniz, 1649-028 Lisbon, Portugal

## Abstract

Chemotherapy-induced nausea and vomiting (CINV) is still a common and debilitating side effect despite recent advances in its prevention and treatment. The intrinsic emetogenicity of chemotherapy agents allowed grouping into four risk groups (high, moderate, low, and minimal risk of emetogenicity). The prevention of acute and delayed CINV for intravenous agents and one day regimens is well studied, although, there are few data about management of CINV induced by oral cytotoxic agents and targeted therapies, usually administered in extended regimens of daily oral use. Until now treatment of nausea and vomiting caused by oral antineoplastic agents remains largely empirical. The level of evidence of prophylactic antiemetics recommended for these agents is low. There are differences in the classification of emetogenic potential of oral antineoplastic agents between the international guidelines and different recommendations for prophylactic antiemetic regimens. Herein we review the evidence for antiemetic regimens for the most used oral antineoplastic agents for solid tumors and propose antiemetic regimens for high to moderate risk and low to minimal risk of emetogenicity.

## 1. Introduction

Chemotherapy-induced nausea and vomiting (CINV) is still a common and debilitating side effect despite recent advances in its prevention and treatment.

At the 2004 Perugia Antiemetic Consensus Guideline meeting, an expert panel used best available data to establish rankings of emetogenicity. The anticancer therapy was divided into four emetic risk groups: high (>90%), moderate (30–90%), low (10–30%), and minimal (<10%) [1]. These percentages represent the number of patients that will experience emesis after the administration of chemotherapeutic agents if no effective antiemetic prophylaxis has been given. The emetogenic potential of the chemotherapeutic agents used is the main risk factor for the degree of CINV [2] and the one that influences the choice of antiemetic prophylaxis. The other risk factors that can be present are young age, female gender, not having a high alcohol intake, experience of emesis during pregnancy, impaired quality of life, and previous experience with chemotherapy [2, 3].

The methodology for this review article was based on an electronic search of the PubMed database to obtain key literature in prevention of nausea and vomiting in patients undergoing oral anticancer therapies for solid tumors in the last 10 years. There was also evaluation of the summary of product characteristics for each oral antineoplastic agent mentioned and clinical trials that referred to the antiemetic prophylaxis used and the results in the prevention of nausea and vomiting.

## 2. Antineoplastic Oral Agents Emetogenicity

Oral chemotherapeutic agents are evaluated separately from intravenous agents, because of intrinsic differences in emetogenicity as well as differing schedules of administration [1, 4]. Emetogenic classification has been established based on that of a full course of oral antineoplastic therapy as clinically employed [4].

International guidelines such as MASCC, ESMO, and NCCN guidelines give recommendations for antiemetic prophylaxis according to the grade of emetogenicity of oral antineoplastic agents. Although there are no prospective clinical trials that can be used to recommend prophylactic antiemetics for oral antineoplastic drugs, all recommendations are based on expert consensus and low levels of evidence [5]. Recommendations based on high levels of evidence are available only for intravenous agents.

The tables referring to emetogenic potential of oral antineoplastic agents in MASCC and ESMO guidelines published in 2010 are slightly different from NCCN guidelines of 2014 (Tables 1 and 2).

In MASCC and ESMO guidelines oral antineoplastic agents are classified into four risk groups as mentioned above and in NCCN guidelines these agents are grouped into moderate to high risk and minimal to low risk groups.

Also, in NCCN guidelines, there are mentioned agents not referred to in MASCC and ESMO guidelines, just like crizotinib, estramustine, lomustine, mitotane, and vismodegib in moderate to high risk group. Crizotinib and vismodegib were approved by European Medicines Agency (EMA), respectively, in 2012 and 2013, after MASCC and ESMO guidelines had been published.

Oral vinorelbine is mentioned in moderate risk group in MASCC and ESMO guidelines and is not mentioned in NCCN guidelines. Oral vinorelbine is not available in the United States.

Imatinib is classified as moderate risk in MASCC and ESMO guidelines and as minimal to low risk in NCCN guidelines.

Etoposide is classified as low risk in MASCC and ESMO guidelines and as moderate to high risk in NCCN guidelines.

Cyclophosphamide and temozolomide are classified as moderate risk in MASCC and ESMO guidelines and as moderate to high risk or minimal to low risk in NCCN guidelines according to the daily dosage and the association of temozolomide with radiotherapy.

Axitinib, cabozantinib, dabrafenib, pazopanib, regorafenib, trametinib, vandetanib, and vemurafenib are classified as minimal to low risk group in NCCN guidelines and are not mentioned in MASCC and ESMO guidelines because EMA approval occurred between 2010 and 2014, after publication of these guidelines.

These small differences between these guidelines may be due to little variations in the experience and expertise of the panel members that collaborated for each one [6]. The NCCN guidelines are more recent and more frequently updated but the bibliographic references are identical to MASCC and ESMO guidelines [4]. Considerations about the dose of chemotherapeutic agents are only made on NCCN guidelines for cyclophosphamide and temozolomide, suggesting that, for lower doses of these drugs, the recommended antiemetic prophylaxis for moderate risk of emetogenicity may be excessive.

Usually CINV is classified as acute, delayed, or anticipatory, and these distinctions have important implications for patient management [7]. Although with extended regimens of daily oral use the distinction between acute and delayed emesis is less clear, cumulative emesis must be considered, as some of the newer agents may only become emetogenic after a week or more of continuous administration [4, 5].

## 3. Antiemetics

There are three categories of drugs most efficient for the management of CINV: type three 5-hydroxytryptamine (5-HT3) receptor antagonists, neurokinin-1 receptor (NK1R) antagonists, and glucocorticoids (especially dexamethasone) [7]. These agents are used alone (glucocorticoids) or in combinations depending on the specific chemotherapy regimen being administered, as recommended in the MASCC and ESMO guidelines and in the ASCO guidelines [1, 8].

The prevention of acute and delayed CINV for intravenous agents and one day regimens is well studied [1, 8], although there is little data about management of CINV caused by oral cytotoxic agents and targeted therapies, usually administered in extended regimens of daily oral use.

NCCN guidelines recommend antiemetic prophylaxis for the following oral agents, classified as having high or moderate emetic risk: hexamethylmelamine, crizotinib, cyclophosphamide (≥100 mg/m^2^/day), estramustine, etoposide, lomustine (single day), mitotane, procarbazine, temozolomide (>75 mg/m^2^/day or ≤75 mg/m^2^/day with concurrent radiotherapy), and vismodegib. The antiemetics recommended are oral 5-HT3 antagonists (such as dolasetron 100 mg po daily, granisetron 2 mg po daily, or 1 mg po bid or ondansetron 16–24 mg po daily) with or without lorazepam (0.5–2 mg po or sublingual every 4 or every 6 h on as needed basis) and with or without either an H2 blocker or a proton pump inhibitor. These antiemetics should be started before chemotherapy and continue daily during chemotherapy. For oral agents of low or minimal emetic risk no routine premedication is required and recommended antiemetics include oral 5-HT3 antagonists, metoclopramide, prochlorperazine, or haloperidol with or without lorazepam and with or without either an H2 blocker or a proton pump inhibitor on as needed basis only [6].

There is no data incorporating NK1 receptor antagonists with oral regimens [6].

These antiemetic recommendations apply to oral chemotherapy only. When combined with intravenous agents in a combination chemotherapy regimen, the antiemetic recommendations for the agent with the highest level of emetogenicity should be followed.

## 4. Antineoplastic Agents with High Emetogenic Potential

There are two oral chemotherapy agents with high emetogenic potential, being rarely used in clinical practice [1, 6].

Hexamethylmelamine (also known as altretamine) is an alkylating agent that may be used in persistent or recurrent epithelial ovarian cancer. The recommended dosage is 260 mg/m^2^/day in 4 divided doses for 14 or 21 days of a 28-day cycle.

Procarbazine is also an alkylating agent used in central nervous system tumors. The PCV regimen includes procarbazine 60 mg/m^2^ on days 8 to 21 every 6 weeks (in combination with lomustine and vincristine) for 6 cycles or 75 mg/m^2^ on days 8 to 21 every 6 weeks (also in combination with lomustine and vincristine) for up to 4 cycles.

NCCN recommendations for antiemetic prophylaxis for moderate to high emetogenic risk may be followed for these agents.

## 5. Antineoplastic Agents with Moderate Emetogenic Potential

The moderate risk category covers a wide range of frequencies of emesis from 30% to 90%. The period of risk lasts for at least 2 days after the last dose of chemotherapy [6].

The agents included in this category are cyclophosphamide, temozolomide, vinorelbine, and imatinib according to MASCC and ESMO guidelines.

According to NCCN guidelines other agents are also included such as crizotinib, estramustine, etoposide, lomustine (single day), mitotane, and vismodegib.

Cyclophosphamide is an alkylating agent. The oral cyclophosphamide is used in adjuvant breast cancer regimens just like cyclophosphamide, methotrexate, and fluorouracil (100 mg/m^2^/day on days 1 to 14 every 28 days for 6 cycles). In this regimen an antiemetic protocol for high/moderate emetogenic chemotherapy is recommended [9]. The British Columbian Cancer Agency (BCCA) recommends ondansetron 8 mg po and dexamethasone 8–20 mg po on D1 of chemotherapy and dexamethasone 4 mg po in the evening of D1 and then 4 mg po bid on D2-D3/4. Prochlorperazine 10 mg po or metoclopramide 10–40 mg po could be used on as needed basis [10]. In 1993, Buser et al. showed that ondansetron given orally, 8 mg three times a day for 15 days, was safe and effective in the control of emesis induced by oral multiple-day cyclophosphamide treatment in breast cancer patients receiving chemotherapy with CMF [11].

Temozolomide is an alkylating agent used in malignant brain tumors and in malignant melanoma (with brain metastasis). Patients with newly diagnosed malignant gliomas treated with concomitant temozolomide (75 mg/m^2^ po daily for 6 weeks) and RT should be medicated with ondansetron 8 mg given 30 minutes prior to first dose of temozolomide and then prochlorperazine 10 mg po 30 minutes prior to each subsequent dose of temozolomide [10]. For adjuvant and palliative temozolomide (150 mg/m^2^ once daily for 5 days every 28 days) the recommended premedication is ondansetron 8 mg po 30 minutes prior to each dose of temozolomide [12, 13]. Palonosetron has been tested in a phase II study in the adjuvant treatment of glioblastoma patients. A single dose of palonosetron 0.25 mg iv before the initiation of multiple oral doses of temozolomide, in patients on treatment with steady doses of dexamethasone, provides a good protection against CINV throughout the overall phase (0–168 h) [14]. In malignant melanoma (temozolomide 200 mg/m^2^/day for 5 days every 28 days) the premedication is also ondansetron 8 mg po 30 minutes prior to each dose of temozolomide [15].

Vinorelbine is a mitotic inhibitor. Oral vinorelbine is used in non-small-cell lung cancer and in breast cancer in mono- or polychemotherapy. In monotherapy the starting dose is 60 mg/m^2^ given on days 1 and 8 of a 3-week cycle and thereafter 80 mg/m^2^ on D1 and D8. It may induce nausea or vomiting because of local gastrointestinal irritation, so a primary prophylaxis with 5-HT3 receptor antagonist 30 min before each intake is recommended [16]. In the associations with cisplatin or taxanes the antiemetic regimens are chosen based on these drugs because of their highest emetic risk. In association with capecitabine or trastuzumab, the prophylaxis could be done with 5-HT3 receptor antagonist before vinorelbine intake.

Imatinib is a tyrosine kinase inhibitor. In GIST (adjuvant and metastatic) it is used as 400 mg once daily but may be increased up to 800 mg daily (400 mg twice daily) in disease progression [17, 18]. In MASCC and ESMO guidelines it is considered as having moderate emetogenic potential and BCCA recommends antiemetic protocol for low/moderate emetogenic chemotherapy. In NCCN guidelines it is classified as minimal to low risk of emetogenicity and so no prophylaxis of emesis is recommended.

Crizotinib is an anaplastic lymphoma kinase (ALK) tyrosine kinase inhibitor used in treatment of metastatic ALK-positive non-small-cell lung cancer. It is administered as 250 mg twice daily. BCCA does not recommend antiemetic premedication [19]. In the product monograph it is mentioned that median time to the onset for nausea and vomiting is 2 to 3 days and most events are mild to moderate in severity and declined in frequency after 3 to 4 weeks of treatment. In clinical trials, the most commonly used antiemetic medications were ondansetron and prochlorperazine [20].

Estramustine is an alkylating agent used in hormone refractory and metastatic prostate cancer. The recommended dosage is 14 mg/kg/day (range: 10–16 mg/kg/day) in 3 or 4 divided doses [21]. Transient nausea and vomiting may occur during the first two weeks of therapy [22].

Etoposide is a topoisomerase II inhibitor. It is classified as having low emetogenic risk by MASCC and ESMO guidelines and as moderate to high risk by NCCN guidelines. It is used in small cell lung cancer (first-line in combination, second-line alone or in combination), non-small-cell lung cancer (alone or in combination), and testicular cancer (in combination, oral therapy for refractory disease). The usual dosage is 100 to 200 mg/m^2^/day for 5 days. If daily doses are >200 mg they should be administered in 2 divided doses. Dosage must be modified to take into account the myelosuppressive effects of other drugs in the combination. The product monograph mentioned that the severity of nausea and vomiting due to oral etoposide is generally mild to moderate with treatment discontinuation required in 1% of patients. Symptoms can usually be controlled with standard antiemetic therapy [23, 24]. The emetic potential of oral etoposide was studied by Einhorn and Brames in 16 patients with refractory germ cell cancer who received daily oral etoposide 50 mg/m^2^ for 21 consecutive days every 4 weeks [25]. None of the patients received prophylactic antiemetics, 11 of 16 had no nausea or vomiting, and only two patients required antiemetic support, being treated with lorazepam and lorazepam with prochlorperazine. The authors suggested that daily oral etoposide does not require prophylactic antiemetics.

Lomustine is an alkylating agent (nitrosourea). It is used in the treatment of recurrent malignant brain tumors. The usual dosage is 130 mg/m^2^ (single dose at fasting or at bedtime) once every 6 weeks. Nausea and vomiting may occur 3 to 6 hours after administration and the duration is generally <24 hours [26]. BCCA suggests ondansetron 8 mg po plus dexamethasone 12 mg po 30 min before lomustine and then dexamethasone 4 mg po twice daily during 24 hours [27].

Mitotane is an adrenal cytotoxic agent. It is used in adrenocortical carcinoma. The initial dose is 2 to 6 g daily in 3 to 4 divided doses, and then it is increased incrementally to 8 to 10 g daily in 3 to 4 divided doses. BCCA recommends prophylactic antiemetic regimens for agents with low/moderate emetic risk [10].

Vismodegib is a hedgehog pathway inhibitor. It is used in basal cell cancer (metastatic or locally advanced), 150 mg once daily. BCCA suggests an antiemetic protocol for low emetogenicity (dexamethasone 4–12 mg po), but antiemetics are not usually required [28].

## 6. Antineoplastic Agents with Low or Minimal Emetogenic Potential

There are even less clinical trials of antiemetic prophylaxis or treatment of emesis associated with agents of low or minimal emetogenic risk. This creates risk of overtreatment if prophylactic regimens for drugs of moderate emetic potential are used [3].

For these agents NCCN guidelines recommend no routine antiemetic premedication and suggest oral 5-HT3 antagonists, metoclopramide, prochlorperazine, or haloperidol with or without lorazepam and with or without either an H2 blocker or a proton pump inhibitor on as needed basis only [6]. If nausea and vomiting occurs, single agent antiemetics can be used in subsequent cycles.

When low or minimal emetic risk oral agents are combined with intravenous agents the antiemetic recommendations for the agent with the highest level of emetogenicity should be followed.

## 7. Management of Emetogenicity of Oral Regimens: Proposed Scheme

Considering the differences in the classification of emetogenic potential of oral antineoplastic agents and the prophylactic antiemetic regimens recommended by the international guidelines, we propose the following recommendations.

*General Recommendations*

- The anticancer agent must be taken with food, unless it is specified differently in summary of product characteristics.
- Choose the antiemetic regimens according to the emetogenic potential of the antineoplastic agent.
- If the antiemetic regimen chosen is insufficient, associate an antiemetic from a different category.
- Check for possible interactions between each antineoplastic agent and the proposed antiemetic regimens.

*Antiemetic Regimens.* For oral antineoplastic agents with high or moderate emetic risk we suggest antiemetic prophylaxis with oral 5-HT3 antagonists, such as ondansetron 8–16 mg 30 minutes before the antineoplastic agent or 8 mg bid during the days in which the oral antineoplastic is administered plus one or two days after it is ended. It may be associated with a glucocorticoid as dexamethasone 4–8 mg 30 minutes before the antineoplastic agent or 2–4 mg bid during oral chemotherapy. The glucocorticoid is especially useful with antineoplastic agents administered one time each week (e.g., vinorelbine). Olanzapine 10 mg once daily may be associated with continuous oral regimens (see the following list). 

*High to Moderate Emetic Risk: Emesis Prevention*

 Start before chemotherapy and continue daily:
 (i) 5-HT3 antagonist: ondansetron 8 mg po bid,
 (ii) ±glucocorticoid: dexamethasone 2–4 mg po bid,
 (iii) ±olanzapine 10 mg po id.

For oral agents of low or minimal emetic risk no routine antiemetic premedication is required and if necessary domperidone 10 mg 3-4 times daily or metoclopramide 10 mg 3-4 times daily with or without lorazepam 0.5–2 mg every 4–6 hours as needed is recommended (see the following list).

*Low to Minimal Emetic Risk: Emesis Prevention*

 No antiemetic prophylaxis
 
*Or*
 (i) domperidone 10 mg po 3-4 times daily,* or*
 (ii) metoclopramide 10 mg po 3-4 times daily,
  (iii) ±lorazepam 0.5–2 mg every 4–6 hours as needed.

## 8. Differential Diagnosis for Emesis in Patients under Oral Antineoplastic Treatment

The oral antineoplastic agents can be responsible for nausea and vomiting in patients under treatment, but with the exception of some drugs previously mentioned, most of these drugs are relatively well tolerated.

So, other causes should be sought in these patients. A meticulous history and physical examination should be performed. Symptom duration (acute versus chronic), frequency, temporal relationship with the oral antineoplastic agents or other drugs, severity, and the characteristics of vomiting episodes and associated symptoms must be characterized. In some circumstances the etiology can be multifactorial. Most frequent disorders associated with nausea and vomiting are listed in the following list.

*Differential Diagnosis for Emesis in Patients under Oral Antineoplastic Treatment*

 (i) Tumor related causes are as follows:
   (a) malignant mechanical obstruction (bowel obstruction, gastric obstruction, and extrinsic compression by hepatomegaly or ascites);
   (b) increased intracranial pressure: primary or secondary brain tumors;
   (c) metabolic abnormalities: hypercalcemia, hyponatremia, and adrenal insufficiency.
 (ii) Treatment related causes are as follows:
   (a) chemotherapy induced;
   (b) anticipatory nausea and vomiting;
   (c) radiotherapy induced;
   (d) postoperative nausea and vomiting.
 (iii) Organic causes (generally not tumor related) are as follows:
   (a) acute gastritis/gastroenteritis;
   (b) gastroesophageal reflux disease;
   (c) functional gastrointestinal disorders;
   (d) acute cholecystitis and acute pancreatitis;
   (e) constipation;
   (f) chronic intestinal pseudo-obstruction;
   (g) vestibular dysfunction;
   (h) myocardial ischemia;
   (i) uremia;
   (j) diabetic ketoacidosis;
   (k) hyperparathyroidism, hypoparathyroidism, and hyperthyroidism;
   (l) sepsis;
   (m) hemorrhage intracranial and abscess intracranial;
   (n) migraine;
   (o) pregnancy;
   (p) psychiatric disease.
 (iv) Drug-induced causes (may not be tumor related) are as follows:
   (a) analgesics: opioids, tramadol, nonsteroidal anti-inflammatory drugs, aspirin;
   (b) antidepressants: selective serotonin reuptake inhibitors and bupropion;
   (c) anticonvulsants;
   (d) iron supplements;
   (e) antibiotics/antivirals: erythromycin, tetracycline, sulfonamides, and acyclovir;
   (f) cardiovascular medications: digoxin, antiarrhythmics, antihypertensives, and oral antidiabetics.

## 9. Conclusions

In clinical practice, for patients on treatment with oral antineoplastic agents, each emetic event represents a distressing symptom that could alter the absorption of chemotherapy with potentially negative effects in terms of efficacy. Until now almost no prospective data is available to guide the use of antiemetic agents in such cases.

We reviewed the evidence for antiemetic regimens used for the most commonly used oral antineoplastic agents for solid tumors and propose antiemetic regimens for agents of high to moderate risk and low to minimal risk of emetogenicity.

Treatment of nausea and vomiting caused by oral antineoplastic agents still remains largely empirical and we hope for advances in management of emetogenicity of oral regimens for solid tumors.

## Figures and Tables

**Table 1:** Emetogenic potential of oral antineoplastic agents most used in solid tumors (based on MASCC and ESMO guidelines 2010).

MASCC and ESMO guidelines 2010	
Degree of emetogenicity (incidence)	Agent
High (>90%)	Hexamethylmelamine
	Procarbazine

Moderate (30–90%)	Cyclophosphamide
	Temozolomide
	Vinorelbine
	Imatinib

Low (10–30%)	Capecitabine
	Etoposide
	Sunitinib
	Everolimus
	Lapatinib

Minimal (<10%)	Methotrexate
	Gefitinib
	Erlotinib
	Sorafenib

**Table 2:** Emetogenic potential of oral antineoplastic agents most used in solid tumors (based on NCCN guidelines 2014).

NCCN guidelines 2014	
Degree of emetogenicity (incidence)	Agent
Moderate to high	Hexamethylmelamine
	Crizotinib
	Cyclophosphamide (≥100 mg/m^2^/day)
	Estramustine
	Etoposide
	Lomustine (single day)
	Mitotane
	Procarbazine
	Temozolomide (>75 mg/m^2^/day)
	Vismodegib

Minimal to low	Axitinib
	Cabozantinib
	Capecitabine
	Cyclophosphamide (<100 mg/m^2^/day)
	Dabrafenib
	Erlotinib
	Everolimus
	Gefitinib
	Imatinib
	Lapatinib
	Methotrexate
	Pazopanib
	Regorafenib
	Sorafenib
	Sunitinib
	Temozolomide (<75 mg/m^2^/day)
	Topotecan
	Trametinib
	Vandetanib
	Vemurafenib

## References

[1] Roila F., Herrstedt J., Aapro M. (2010). Guideline update for MASCC and ESMO in the prevention of chemotherapy and radiotherapy-induced nausea and vomiting: results of the Perugia consensus conference. *Annals of Oncology*.

[2] Jordan K., Sippel C., Schmoll H.-J. (2007). Guidelines for antiemetic treatment of chemotherapy-induced nausea and vomiting: past, present, and future recommendations. *The Oncologist*.

[3] Olver I., Clark-Snow R. A., Ballatori E., Espersen B. T., Bria E., Jordan K. (2011). Guidelines for the control of nausea and vomiting with chemotherapy of low or minimal emetic potential. *Supportive Care in Cancer*.

[4] Grunberg S. M., Warr D., Gralla R. J. (2011). Evaluation of new antiemetic agents and definition of antineoplastic agent emetogenicity—state of the art. *Supportive Care in Cancer*.

[5] Hesketh P. J. (2008). Chemotherapy-induced nausea and vomiting—review article. *The New England Journal of Medicine*.

[6] Ettinger D., Berger M., Armstron D.

[7] Hesketh P. (2014). Prevention and treatment of chemotherapy-induced nausea and vomiting. *UpToDate*.

[8] Basch E., Prestrud A. A., Hesketh P. J. (2011). Antiemetics: american Society of Clinical Oncology clinical practice guideline update. *Journal of Clinical Oncology*.

[9] Ellard S.

[10] Hoskins P. (2014). *BBCA Guidelines for Prevention and Treatment of Chemotherapy-Induced Nausea and Vomiting in Adults*.

[11] Buser K. S., Joss R. A., Piquet D. (1993). Oral ondansetron in the prophylaxis of nausea and vomiting induced by cyclophosphamide, methotrexate and 5-fluorouracil (CMF) in women with breast cancer. Results of a prospective, randomized, double-blind, placebo-controlled study. *Annals of Oncology*.

[12] Thiessen B. (2014). BCCA protocol summary for concomitant (dual modality) and adjuvant temozolomide for newly diagnosed malignant gliomas with radiation. *BC Cancer Agency Protocol Summary CNAJTZRT*.

[13] Thiessen B.

[14] Rozzi A., Nardoni C., Corona M. (2011). Palonosetron for the prevention of chemotherapy-induced nausea and vomiting in glioblastoma patients treated with temozolomide: a phase II study. *Supportive Care in Cancer*.

[15] Klassa R.

[16] Aapro M., Finek J. (2012). Oral vinorelbine in metastatic breast cancer: a review of current clinical trial results. *Cancer Treatment Reviews*.

[17] Knowling M.

[18] Knowling M. BCCA Protocol Summary for Treatment of Advanced C-Kit Positive and C-Kit Negative Gastrointestinal Stromal Cell Tumours (GISTs) Using Imatinib (GLEEVEC).

[19] Lee C.

[20] Pfizer Canada Inc (2014). *Crizotinib (XALKORI) Product Monograph*.

[21] Chi K. BCCA Protocol Summary for Androgen-Independent Prostate Cancer Using Estramustine Phosphate (Interim Version).

[22] Pfizer Canada Inc (2008). *MCYT Product Monograph*.

[23] Murray N. (2014). *BCCA Protocol Summary for Palliative Therapy of Extensive Stage Small Cell Lung Cancer (SCLC) with Oral Etoposide*.

[24] Bristol-Myers Squibb Canada (2008). *Etoposide (VEPESIDE) Product Monograph*.

[25] Einhorn L. H., Brames M. J. (2006). Emetic potential of daily oral etoposide. *Supportive Care in Cancer*.

[26] Bristol-Myers Squibb Canada (2010). *Lomustine (CeeNU) Product Monograph*.

[27] Thiessen B.

[28] Savage K. BCCA Protocol Summary for the Treatment of Metastatic or Locally Advanced Basal Cell Carcinoma Using Vismodegib.

